# Slit2N/Robo1 Inhibit HIV-gp120-Induced Migration and Podosome Formation in Immature Dendritic Cells by Sequestering LSP1 and WASp

**DOI:** 10.1371/journal.pone.0048854

**Published:** 2012-10-31

**Authors:** Anil Prasad, Paula M. Kuzontkoski, Ashutosh Shrivastava, Weiquan Zhu, Dean Y. Li, Jerome E. Groopman

**Affiliations:** 1 Division of Experimental Medicine, Beth Israel Deaconess Medical Center, Harvard Medical School, Boston, Massachusetts, United States of America; 2 Department of Medicine and Molecular Medicine Program, University of Utah, Salt Lake City, Utah, United States of America; Academic Medical Center, The Netherlands

## Abstract

Cell-mediated transmission and dissemination of sexually-acquired human immunodeficiency virus 1 (HIV-1) in the host involves the migration of immature dendritic cells (iDCs). iDCs migrate in response to the HIV-1 envelope protein, gp120, and inhibiting such migration may limit the mucosal transmission of HIV-1. In this study, we elucidated the mechanism of HIV-1-gp120-induced transendothelial migration of iDCs. We found that gp120 enhanced the binding of Wiskott-Aldrich Syndrome protein (WASp) and the Actin-Related Protein 2/3 (Arp2/3) complex with β-actin, an interaction essential for the proper formation of podosomes, specialized adhesion structures required for the migration of iDCs through different tissues. We further identified Leukocyte-Specific Protein 1 (LSP1) as a novel component of the WASp-Arp2/3-β-actin complex. Pretreating iDCs with an active fragment of the secretory glycoprotein Slit2 (Slit2N) inhibited HIV-1-gp120-mediated migration and podosome formation, by inducing the cognate receptor Roundabout 1 (Robo1) to bind to and sequester WASp and LSP1 from β-actin. Slit2N treatment also inhibited Src signaling and the activation of several downstream molecules, including Rac1, Pyk2, paxillin, and CDC42, a major regulator of podosome formation. Taken together, our results support a novel mechanism by which Slit2/Robo1 may inhibit the HIV-1-gp120-induced migration of iDCs, thereby restricting dissemination of HIV-1 from mucosal surfaces in the host.

## Introduction

After sexual contact with HIV-1, immature DCs (iDCs) capture the virus in submucosal tissue and then migrate to lymphoid tissues where they infect CD4^+^ T cells. These initial stages of HIV-1 infection result in rapid dissemination of the pathogen throughout the host [1]–[5]. Impeding the migration of iDCs could provide a novel therapeutic strategy to arrest mucosal transmission of HIV-1. To that end, we assessed Slit2/Robo1, a novel ligand-receptor pathway, as a means to inhibit HIV-1-induced migration of iDCs.

DC function is dependent on the ability of iDCs to migrate through different tissues [6]–[8]. Various migratory stimuli induce cytoskeletal rearrangements in iDCs and promote the formation of one dominant protrusion, or lamellipodium, at the leading edge of the cell, which is oriented in the direction of migration [7]. Non-migrating cells can also generate lamellipodia; however, in contrast to migrating DCs, they produce multiple lamellipodia that protrude at various points around the cell. This prevents the cells from adopting a polar orientation, and results in no net motility [9].

In iDCs, numerous, small, round, adhesion points between the cell and its substratum localize to the leading edge of the cell [7], [10], [11]. Recent studies indicate that the proper formation and localization of these adhesion structures, or “podosomes,” are required for the migration of iDCs through various tissue barriers [7], [12]. Podosomes are characterized by an actin-rich core, which is surrounded by a ring-like structure, consisting primarily of integrins, tyrosine kinases, and adhesion molecules, including vinculin and paxillin [11], [13]. Attachment of iDCs to the substratum via integrins and their binding partners induces receptor tyrosine kinase signaling, specifically through PKC (protein kinase C), Src kinase and Pyk2 (proline-rich tyrosine kinase-2) [13], [14]. Pyk2 activates the Rho GTPase CDC42, which relieves the conformational autoinhibition of WASp, a major regulator of the actin cytoskeleton [10]. WASp can then interact with the Arp2/3 complex and actin to promote actin polymerization and induce the nucleation of actin filaments within the podosome core [11], [15]. Integrin activation can also promote paxillin to mediate the formation of the podosome ring structure [13]; CDC42 and Rac1 signaling contribute to ring formation by activating the adhesion mediator, vinculin, in iDCs [12], [13].

Podosomes are highly dynamic. Their average half-life is between 2 and 12 minutes, during which period the core is refreshed 2 to 3 times [16], [17]. Moreover, the efficient formation of multiple podosomes at the leading edge of a cell, and their disassembly at the rear, are critical for the migration of iDCs [10], [17].

Recent studies implicate a role for Slit2 and its ligand, Robo1, in the migration of several immune cells, including dendritic cells, by modulating cytoskeletal dynamics [18]–[22]. Slit2 belongs to the Slit family of proteins (Slits 1, 2, and 3, in mammals), which are large, extracellular matrix-secreted and matrix-anchored glycoproteins [23]. The Slits were first identified as modulators of neuronal repulsion during development; however, recent studies highlight their role as multifunctional signaling molecules [13], [24], [25].

Robo1 is a single-pass transmembrane receptor, which belongs to the immunoglobulin (Ig) superfamily of cell adhesion molecules [26], [27]. Four Robo proteins (Robos 1–4) have been identified in mammals [28]–[30]. The cytoplasmic domains of the Robo receptors do not possess autonomous catalytic activity; therefore, they must interact with other signaling molecules or co-receptors to exert their functional effects [23], [31]. Robo 1 signaling is primarily initiated upon the binding of Slit2 [29], [32], [33].

To model HIV-induced chemotaxis of iDCs, we used M-tropic HIV-1 envelope protein, M-gp120, and then explored the effects of Slit2 on this migration. Since proper podosome function appears to be necessary for iDC migration, we also examined the effects of M-gp120 and Slit2 on both podosome formation and localization in iDCs, specifically, the complexing of key core podosome-associated proteins, WASp, Arp2/3 and β-actin.

## Results

### Slit2N Inhibits M-gp120-Induced Migration and Podosome Formation in iMDDCs

**Figure 1:**
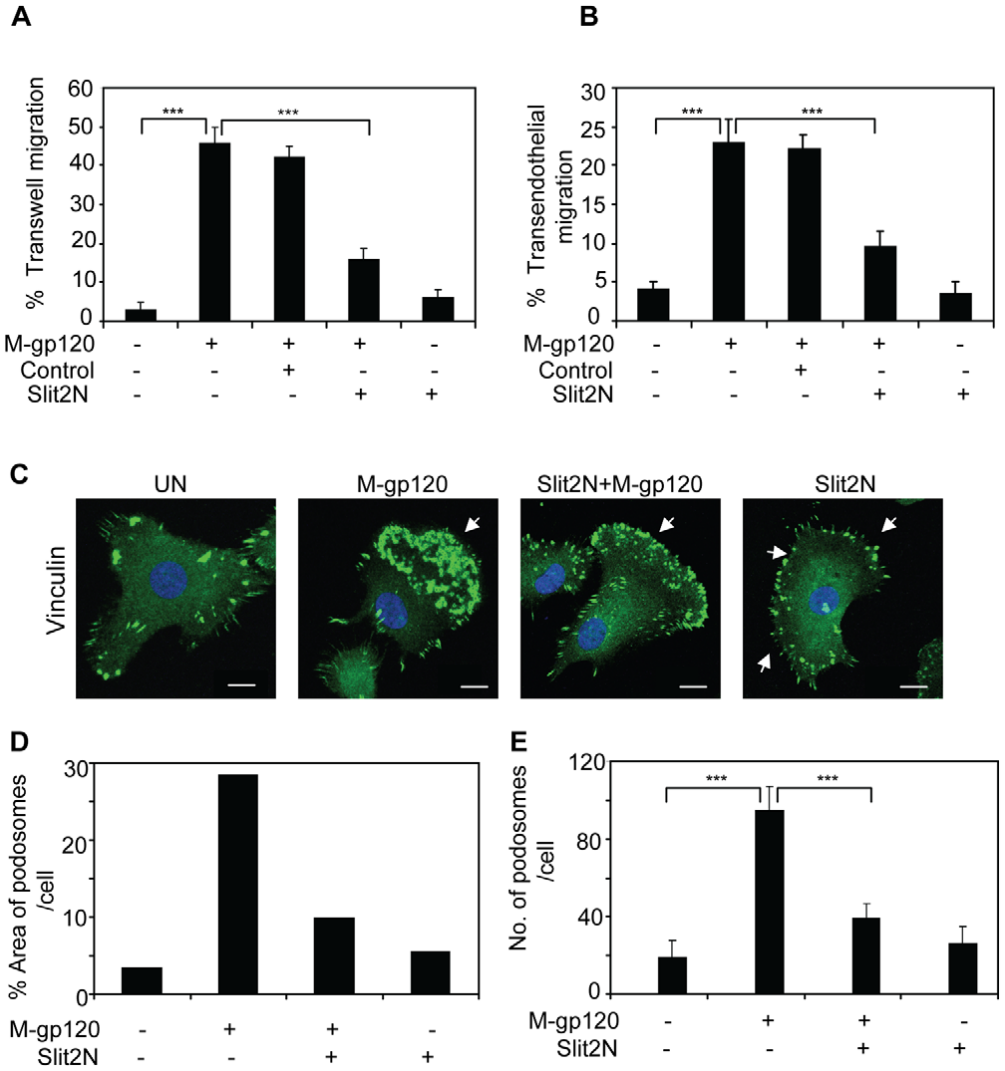
Slit2N inhibits HIV-1-gp120-induced migration and podosome formation of iMDDCs. (**A**) iMDDC transwell migration: cells were incubated with Slit2N, Slit2 negative control, or media alone for 2 hours, then seeded to the upper compartments of transwell chambers. Media +/− M-gp120 or Slit2N was added to the bottom compartments of the chambers. After 3 hours, iMDDCs that had migrated to the lower chambers were counted by hemocytometer. Data indicate the mean ± SD of 5 independent experiments (***p≤0.001). (**B**) iMDDC transendothelial migration: HUVECs were seeded into the upper compartment of transwell chambers and incubated at 37°C, 5% CO_2_, to confluency. iMDDCs were incubated with Slit2N, Slit2 negative control, or media alone for 2 hours, then seeded to the upper compartments of transwell chambers. Media +/− M-gp120 or Slit2N was added to the bottom compartments of the chambers. After 8 hours, iMDDCs that had migrated to the lower chambers were counted by hemocytometer. Data indicate the mean ± SD of 5 independent experiments (***p≤0.001). (**C**) Vinculin expression by confocal microscopy. iMDDCs were cultured on chamber slides and left untreated or incubated with M-gp120, Slit2N then M-gp120, or Slit2N alone (Slit2N incubation: 2 hours; M-gp120 incubation: 1 hour) before fixing and staining cells. Green  =  Vinculin; Blue  =  DAPI. Scale bars  = 2 µm. Arrows indicate a dense accumulation of podosomes at the cell's leading edge (“M-gp120” image), a reduced number of podosomes clustered at a weaker leading edge (“Slit2N + M-gp120” image), and a reduced number of podosomes localized around the cell's periphery (“Slit2N” image). Representative images are shown. (**D**) Percent of cell area covered by podosomes in the slides from Figure 1C, as described in Materials and Methods. (**E**) Number of podosomes per cells as described in Materials and Methods. Cells were treated as above. Data indicate the mean ± SD of 3 independent experiments x 50 cells per experimental condition (***p≤0.001).

M-gp120 can induce the migration of iDCs *in vitro* [34], and Slit2 has been shown to inhibit the migration of various immune cells, including dendritic cells [19]–[22]. To explore whether Slit2 might inhibit iDC migration induced by M-gp120, we pretreated immature, monocyte-derived dendritic cells (iMDDCs) with Slit2N, an active fragment of Slit2 [35], or with a Slit2 negative control [36], and plated the cells in the upper chamber of transwell plates. To the media in the bottom chamber of the transwells, we added M-gp120, Slit2N or PBS. Consistent with prior results [34], M-gp120 enhanced the migration of iMDDCs (Figure 1A). Pretreating the cells with Slit2N significantly inhibited M-gp120-enhanced migration (Figure 1A). To more closely model the environment encountered by migrating dendritic cells, we assessed iMDDC transwell migration through an endothelial cell monolayer using the conditions described above. We observed that M-gp120 greatly enhanced the transendothelial migration of iMDDCs, and that pretreatment with Slit2N significantly inhibited this migration (Figure 1B).

Recent studies suggest a central role for podosomes in the migration of immature dendritic cells and other cell types that cross tissue boundaries [6]–[8]. We hypothesized that M-gp120 may promote iMDDC migration by enhancing the formation of podosomes in iMDDCs, since Slit2 can modulate the actin cytoskeleton [25], [31], [37], a critical component of podosomes. Podosome formation was evaluated using confocal microscopy. iMDDCs pretreated with Slit2N, a negative control, or PBS, were then stimulated with M-gp120 or Slit2N alone. We incubated the treated iMDDCs with an antibody to vinculin, a protein known to localize to the podosome ring structure [7]. M-gp120 significantly enhanced the formation of podosomes, which localized to the leading edge of the cells (Figure 1C, “M-gp120”), a morphology consistent with migration; pretreatment with Slit2N inhibited much of the podosome formation induced by M-gp120 (Figure 1C, “Slit2N + M-gp120”). While treatment with Slit2N alone did not appear to alter the size or number of podosomes, their altered distribution suggested that Slit2N induced the existing podosomes and focal adhesion-like structures to localize to the periphery of the cells in a diffuse, evenly distributed manner (Figure 1C, “Slit2N”). To quantify this change, we measured the percent area covered by podosomes in the cells (with the same images used in Figure 1C) and the number of podosomes per cell, as described in Materials and Methods. The area covered by podosomes increased significantly after treatment with M-gp120, and pretreatment with Slit2N prevented much of this increase (Figure 1D), while treatment with Slit2N alone did not significantly alter the area covered by podosomes (Figure 1D). In addition, the number of podosomes per cell in iMDDCs increased significantly after M-gp120 treatment, and pretreatment with Slit2N inhibited this M-gp120-induced increase (Figure 1E). Again, treatment with Slit2N alone had no significant affect on the number of podosomes (Figure 1E). Taken together, these data indicate that M-gp120 may enhance iMDDC migration by enhancing the number of podosomes per cell and inducing them to cluster to the leading edge of the cells, and that Slit2N can prevent M-gp120-induced migration and transendothelial migration of iMDDCs. Our observations also suggest that Slit2N may inhibit iMDDC migration by depolarizing the cells, and inhibiting the formation and aggregation of podosomes to the leading edge.

### LSP1 Contributes to M-gp120-Induced Migration and Podosome Formation in iMDDCs

**Figure 2:**
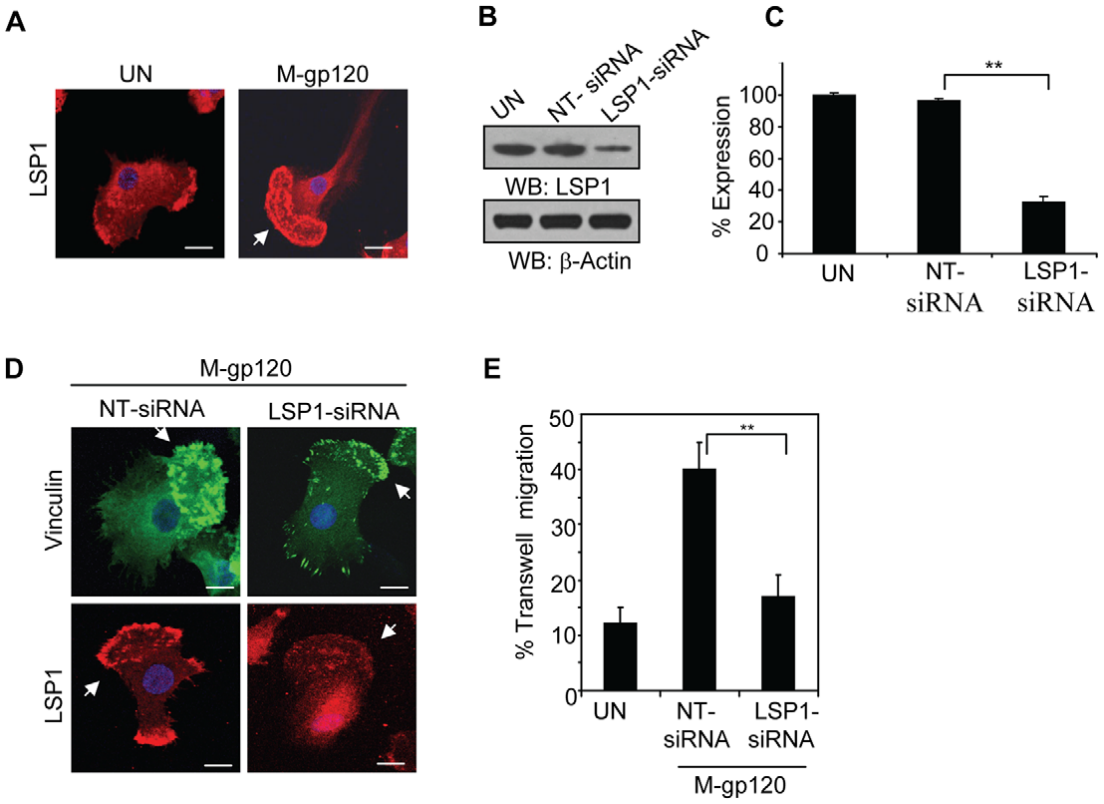
LSP1 contributes to M-gp120-induced migration and podosome formation of iMDDCs. (**A**) LSP1 expression by confocal microscopy. iMDDCs were cultured on chamber slides and left untreated or incubated with M-gp120 for 1 hour before fixing and staining cells. Red  =  LSP1; Blue  =  DAPI. Scale bars  = 2 µm. Arrow indicates the cell's leading edge. Representative images are shown. (**B**) Representative Western blot analysis of LSP1 expression in untreated iMDDCs and in iMDDCs transfected with NT-siRNAs or LSP1-specific siRNAs. β-actin used as loading control. (**C**) Quantitative analysis of LSP1 expression in untreated iMDDCs and in iMDDCs transfected with NT-siRNAs or LSP1-specific siRNAs. The band intensity in each lane of 3 independent Western blots (as in Figure 2B) was determined by densitometry. Percent expression was calculated relative to the untreated control cells, whose LSP1 levels were established as 100%. Data represent the mean ± SD of 3 independent experiments (**p≤0.01). (**D**) LSP1 and vinculin expression by confocal microscopy. iMDDCs transfected with NT-siRNAs or with LSP1-specific siRNAs were cultured on chamber slides and incubated with M-gp120 for 1 hour before fixing and staining cells. Green  =  Vinculin; Red  =  LSP1; Blue  =  DAPI. Scale bars  = 2 µm. Arrows in the upper left panel and lower left panel indicate a dense accumulation of podosomes at the cells' leading edge; while arrows in the upper right panel and lower right panel indicate fewer podosomes, more diffusely distributed throughout cells without a distinct leading edge. Represent­ative images are shown. (**E**) Transwell Migration: iMDDCs and iMDDCs transfected with NT-siRNAs or with LSP1-specific siRNAs were seeded to the upper compartments of transwell chambers. Media +/− M-gp120 was added to the bottom compartments of the chambers. After 2 hours, cells that had migrated to the lower chambers were counted by hemocytometer. Data indicate the mean ± SD of 4 independent experiments (**p≤0.01).

Earlier studies show that in iDCs, M-gp120 can activate LSP1 and promote its binding to actin [34], a major constituent of the podosome core [7], [11], [13]. Moreover, this binding appeared to be critical for iDC migration [34]. To examine the potential role of LSP1 in M-gp120-induced podosome formation in iDCs, we examined by confocal microscopy the expression of LSP1 in untreated iMDDCs and in those incubated with M-gp120. M-gp120 greatly enhanced the localization of LSP1 to podosomes at the leading edge of the cells (Figure 2A, “M-gp120”), suggesting a role in podosome formation and function. To investigate this further, we transiently transfected iMDDCs with non-targeted (NT) siRNAs or LSP1-specific siRNAs, and confirmed the reduction in LSP1 expression by Western blot analysis (Figure 2B). LSP1 expression was quantitated by densitometry. Consistent with the Western blot analysis, iMDDCs transfected with LSP1-specific siRNAs expressed significantly lower levels of LSP1 than those transfected with non-targeted siRNAs (Figure 2C). We treated both groups of transfectants with M-gp120, and examined vinculin (to highlight podosomes) and LSP1 expression by confocal microscopy. M-gp120 induced the formation and leading edge localization of podosomes in the iMDDCs with endogenous LSP1 expression (Figure 2D, upper left panel). M-gp120 also induced the localization of LSP1 to the podosomes at the leading edge of the NT-siRNA transfected cells (Figure 2D, lower left panel). In iMDDCs with reduced LSP1 expression, M-gp120 induced significantly fewer podosomes, and generated a less focused, weaker, leading edge structure than those with endogenous LSP1 expression (Figure 2D, upper right panel). Furthermore, LSP1 was expressed diffusely throughout the cells even after M-gp120 treatment (Figure 2D, lower right panel). These data suggest that sufficient levels of LSP1 may be required for M-gp120-induced podosome formation and their localization to the leading edge of iMDDCs.

To explore the impact of reduced LSP1 expression on M-gp120-mediated dendritic cell function, we compared the M-gp120-induced transwell migration of iMDDCs transfected with NT-siRNAs or with LSP1-specific siRNAs, to unstimulated iMDDCs. M-gp120 significantly enhanced transwell migration of the iMDDCs transfectants with endogenous LSP1 expression (Figure 2E, “NT-siRNA”); however, M-gp120-mediated migration was severely impeded in the transfectants with reduced levels of LSP1 (Figure 2E, “LSP1-siRNA”). These results suggest that sufficient levels of LSP1 are required for M-gp120-enhanced migration of iMDDCs. Taken together, our data suggest that LSP1 plays a role in M-gp120-induced podosome formation and migration of iMDDCs.

### Slit2N Inhibits the M-gp120-Enhanced Association of LSP1 with the WASp-Arp2/3-β-Actin Complex in iMDDC Podosomes

**Figure 3:**
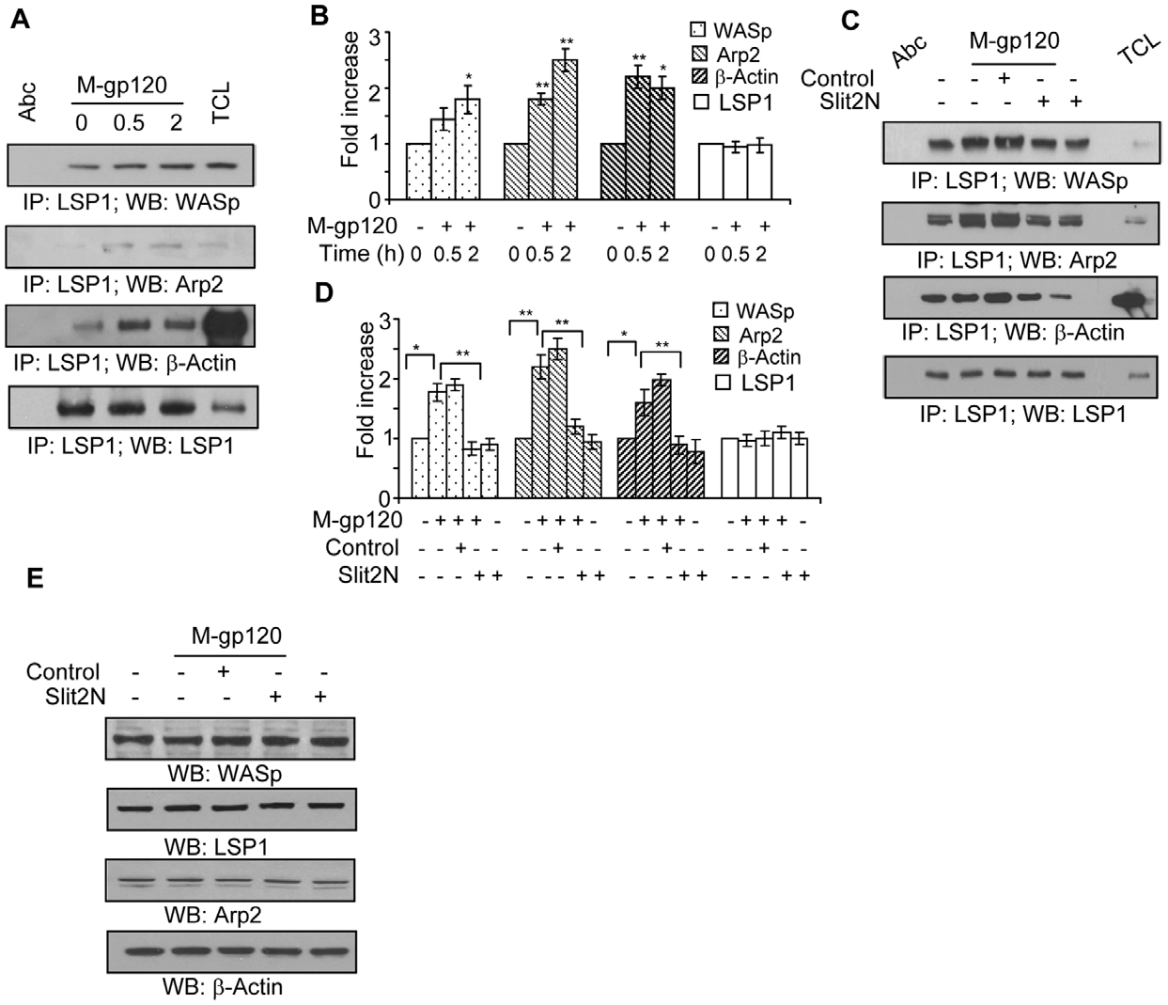
Slit2N inhibits M-gp120-induced association of LSP1, WASp, Arp2/3, and β-actin in iMDDCs. (**A**) Representative immunoprecipitation of LSP1 with WASp, Arp2 and β-actin in iMDDCs incubated for various times with M-gp120. LSP1 used as loading control. (**B**) Quantitative analysis of the immunoprecipitation of LSP1 with WASp, Arp2 and β-actin in iMDDCs incubated for various times with M-gp120. LSP1 used as loading control. The band intensity in each lane was determined by densitometry. The fold change was determined by calculating the value of each lane vs. the unstimulated control (0 h, M-gp120 “−”). Data represent the mean ± SD of 3 independent experiments (*p≤0.05, **p≤0.01). (**C**) Representative immunoprecipitation of LSP1 with WASp, Arp2 and β-actin in untreated iMDDCs, and in iMDDCs incubated with M-gp120, Slit negative control then M-gp120, Slit2N then M-gp120, or Slit2N alone. LSP1 used as loading control. (**D**) Quantitative analysis of the immunoprecipitation of LSP1 with WASp, Arp2 and β-actin in untreated iMDDCs, and in iMDDCs incubated with M-gp120, Slit negative control then M-gp120, Slit2N then M-gp120, or Slit2N alone. LSP1 used as loading control. The band intensity in each lane was determined by densitometry. The fold change was determined by calculating the value of each lane vs. the unstimulated control. Data represent the mean ± SD of 3 independent experiments (*p≤0.05, **p≤0.01). (**E**) Representative Western blot analysis of LSP1, WASp, Arp2 and β-actin in untreated iMDDCs, and in iMDDCs incubated with M-gp120, Slit negative control then M-gp120, Slit2N then M-gp120, or Slit2N alone.

Upon activation, CDC42 interacts with WASp, the Arp2/3 complex, and cytoskeletal actin (β-actin), and induces the nucleation and polymerization of monomeric actin into actin filaments within the podosome core, which promotes podosome formation and iDC migration [38]. In addition, a recent study demonstrated that the binding of LSP1 and actin is important for M-gp120-induced chemotaxis of iDCs [34]. We examined the potential effect of M-gp120 on the binding of LSP1 with the WASp-Arp2/3-β-actin complex in iMDDCs. We collected lysates from untreated iMDDCs, and from those incubated with M-gp120 for 30 minutes and 2 hours. Using LSP1 immunoprecipitation and Western blot analysis, we examined the effects of M-gp120 on the interaction of LSP1 with WASp, Arp2/3, and β-actin (Figures 3A and 3B). M-gp120 enhanced the association LSP1 with all three proteins; moreover, their association with LSP1 increased with time (Figures 3A and 3B). These data suggest that the M-gp120-mediated association of LSP1 with the WASp-Arp2/3-β-actin complex within the podosome core may be important for podosome formation of iMDDCs.

To investigate if Slit2N can inhibit this association, we collected lysates from untreated iMDDCs, and from iMDDCs incubated with M-gp120, Slit2 negative control + M-gp120, Slit2N + M-gp120, and Slit2N alone. After LSP1 immunoprecipitation, we examined by Western blot analysis the interaction of LSP1 with WASp, Arp2/3, and β-actin (Figures 3C and 3D). M-gp120 enhanced the binding of LSP1 to all three proteins, and preincubation with Slit2N inhibited much of the M-gp120-enhanced binding (Figures 3C and 3D). Treatment with Slit2N alone inhibited the association of LSP1 with WASp, Arp2/3, and β-actin as much or more than the combination of Slit2N and M-gp120 (Figures 3C and 3D).

To explore if M-gp120 or Slit2N may affect this association by altering expression levels, we examined by Western blot analysis their expression in iMDDCs under the same conditions. The levels of each of the four proteins in iMDDCs were identical under all conditions investigated (Figure 3E), indicating that neither M-gp120 nor Slit2N affected the expression of LSP1, WASp, Arp2/3 or β-actin in iMDDCs; therefore, M-gp120 and Slit2N must modulate the association among these proteins by an alternative mechanism.

**Figure 4:**
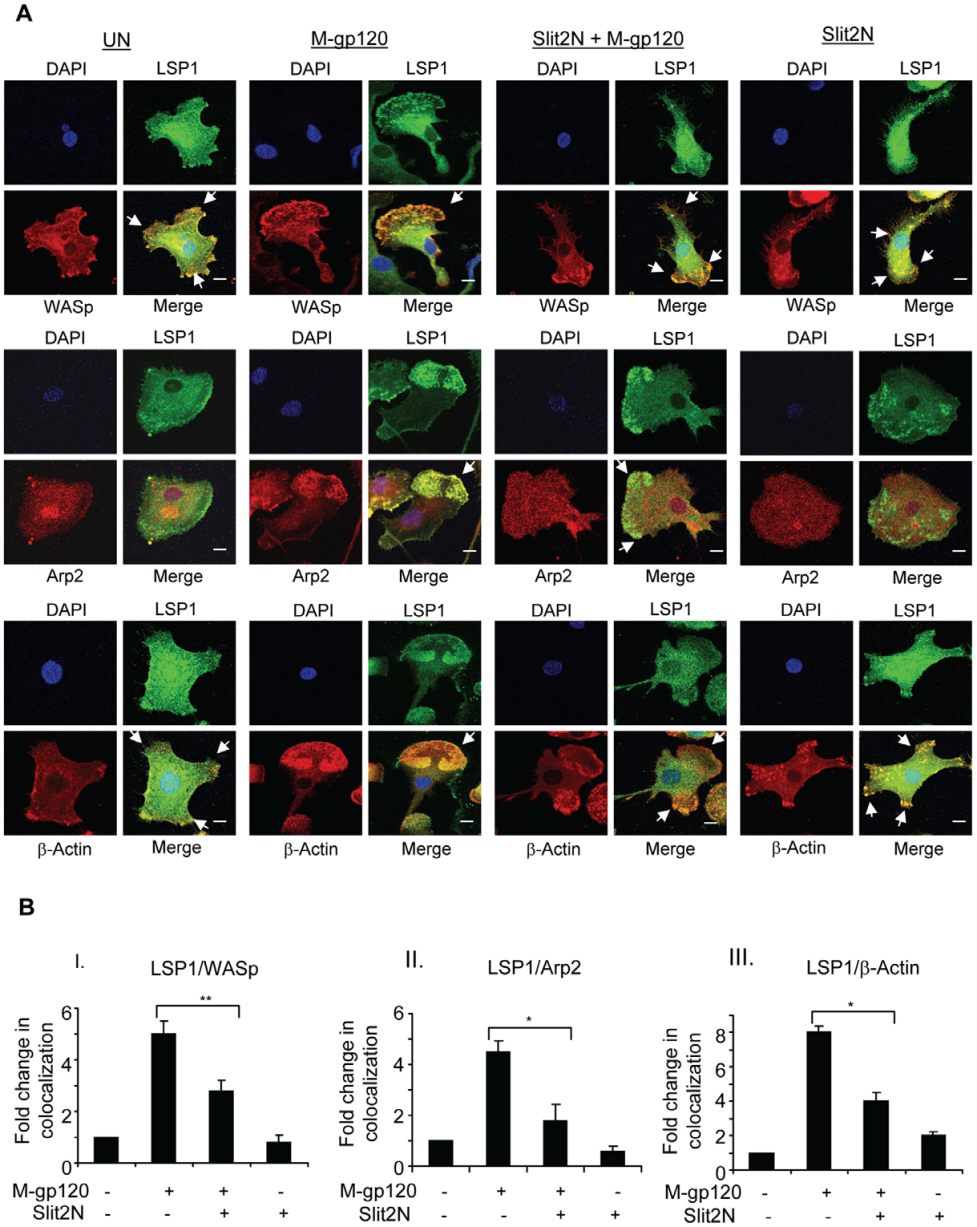
Slit2N inhibits M-gp120-induced colocalization of LSP1, WASp, Arp2/3, and β-actin to iMDDC podosomes. (**A**) Expression and colocalization of LSP1, WASp, Arp2/3 and β-actin by confocal microscopy. iMDDCs were cultured on chamber slides and left untreated or incubated with M-gp120, Slit2N then M-gp120, or Slit2N alone (Slit2N incubation: 2 hours; M-gp120 incubation: 1 hour) before fixing and staining cells. Green  =  LSP1; Red  =  WASp, Arp2/3, or β-actin, as indicated; Yellow/orange  =  merge; Blue  =  DAPI. In the “Merge” images: no arrow indicates a rounded cellular morphology, which is characteristic of non-migrating iMDDCs; one arrow indicates a single lamellipodium, densely populated with podosomes, at the leading edge of a polarized cell, which is characteristic of migrating iMDDCs; multiple arrows indicate multiple lamellipodia/focal adhesions with various orientations, which are characteristic of non-migrating iMDDCs. Scale bars  = 2 µm. Representative images are shown. (**B**) Quantitative analysis of the colocalization of LSP1 with (I) WASp, (II) Arp2/3 and (III) β-actin in iMDDCs, under conditions identical to Figure 4B, using confocal microscopy and Volocity® software. Data represent the mean ± SD of 3 independent experiments x 3 randomly chosen cells per condition (*p≤0.05,**p≤0.01).

To explore this possibility, we examined by confocal microscopy the expression and colocalization of LSP1 with WASp, Arp2/3 and ß-actin in iMDDCs. Untreated iMDDCs displayed multiple lamellipodia or no distinct lamellipodium, morphologies consistent with lack of motility (Figure 4A, “UN” panels). In these cells, LSP1 colocalized moderately with WASp and β-actin at the cell periphery, in multiple lamellipodia (Figure 4A); however, Arp2/3 was diffusely expressed and did not appear to colocalize with LSP1 (Figure 4A). After treatment with M-gp120, the cells became polarized, developed a distinct, single lamellipodium, and podosome numbers increased and localized to the cells' leading edge, morphologies consistent with migration (Figure 4A, “M-gp120” panels). In these cells, LSP1 strongly colocalized with WASp, Arp2/3, and β-actin in the podosomes at the leading edge of iMDDCs (Figure 4A, “M-gp120” panels). When cells were pretreated with Slit2N before incubation with M-gp120, they were less polarized, the leading edge was less distinct, or multiple lamellipodia replaced the single leading edge (Figure 4A, “Slit2N + M-gp120” panels). The colocalization of LSP1 with WASp and β-actin to the lamellipodia was weaker, and again, Arp2/3 was diffusely distributed throughout the cell with little or no colocalization with LSP1 (Figure 4A, “Slit2N + M-gp120” panels). After treatment with Slit2N alone, cell morphology and the colocalization of LSP1 with WASp, Arp2/3 and β-actin was very similar to that of untreated iMDDCs (Figure 4A, “Slit2N” panels). (Note: merged images at a lower magnification are provided in Supporting Information (Figure S1) to demonstrate that the images displayed in Figure 4A are representative of the overall cultures.) To quantitate these colocalization data we used Volocity® software as described in Materials and Methods. Briefly, colocalization of proteins in untreated cells was considered “1,” and fold change in protein colocalization in the treated cells was calculated relative to the untreated control. Consistent with the confocal images, M-gp120 enhanced the colocalization of LSP1 with WASp, Arp2/3, and β-actin; pretreatment with Slit2N significantly inhibited this M-gp120-induced colocalization; and colocalization of the proteins in cells treated with Slit2N alone was very similar to that of untreated iMDDCs (Figure 4B). Our results indicate that M-gp120 induced the colocalization of LSP1 with WASp, Arp2/3 and β-actin to the podosomes at the leading edge of migrating iMDDCs, and that Slit2N inhibited this colocalization. These data suggest that M-gp120 and Slit2N may enhance or inhibit, respectively, podosome formation and iMDDC migration, by modulating the association of LSP1 with WASp, Arp2/3 and β-actin.

### Slit2N Alters the Localization and Binding of Robo1 to WASp and LSP1 in M-gp120-Treated iMDDCs

**Figure 5:**
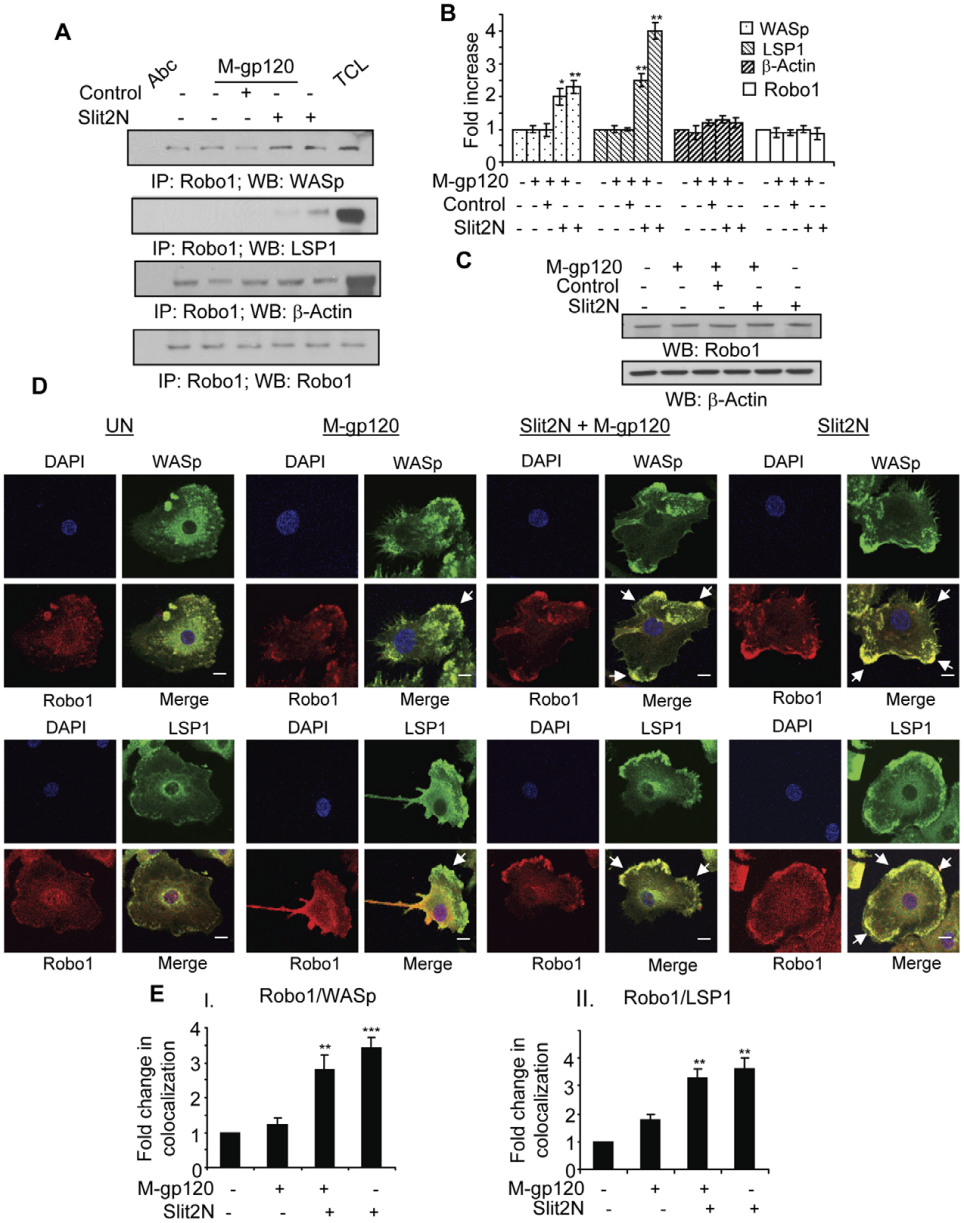
Slit2N enhances Robo1, WASp, and LSP1 association, and their colocalization around the periphery of iMDDCs. (**A**) Representative immunoprecipitation of Robo1 with WASp, LSP1, and β-actin in untreated iMDDCs, and in iMDDCs incubated with M-gp120, Slit negative control then M-gp120, Slit2N then M-gp120, or Slit2N alone. Robo1 used as loading control. (**B**) Quantitative analysis of the immunoprecipitation of Robo1 with WASp, LSP1, and β-actin in untreated iMDDCs, and in iMDDCs incubated with M-gp120, Slit negative control then M-gp120, Slit2N then M-gp120, or Slit2N alone. Robo1 used as loading control. The band intensity in each lane was determined by densitometry. The fold change was determined by calculating the value of each lane vs. the unstimulated control. Data represent the mean ± SD of 3 independent experiments (*p≤0.05, **p≤0.01). (**C**) Representative Western blot analysis of Robo1 expression in untreated iMDDCs, and in iMDDCs incubated with M-gp120, Slit2 negative control then M-gp120, Slit2N then M-gp120, or Slit2N alone. β-actin used as loading control. (**D**) Expression and colocalization of Robo1, WASp, and LSP1 by confocal microscopy. iMDDCs were cultured on chamber slides and left untreated or incubated with M-gp120, Slit2N then M-gp120, or Slit2N alone (Slit2N incubation: 2 hours; M-gp120 incubation: 1 hour) before fixing and staining cells. Red  =  Robo1; Green  =  WASp or LSP1, as indicated; Yellow/orange  =  merge; Blue  =  DAPI. In the “Merge” images: no arrow indicates a rounded cellular morphology with diffuse or no colocalization of the proteins; one arrow indicates a single lamellipodium, but little or no colocalization of proteins to the podosomes at the leading edge of the iMDDC; multiple arrows indicate colocalization of proteins around the periphery of the cells, or in multiple lamellipodia with various orientations. Scale bars  = 2 µm. Representative images are shown. (**E**) Quantitative analysis of the colocalization of Robo1 with (I) WASp and (II) LSP1 in iMDDCs, under conditions identical to Figure 5D, using confocal microscopy and Volocity® software. Data represent the mean ± SD of 3 independent experiments x 3 randomly chosen cells per condition (**p≤0.01, ***p≤0.001 for cells treated with Slit2N, then M-gp120 or just Slit2N vs. untreated control).

The signaling between Slit2 and its binding partner, Robo 1, inhibits glioma cell migration *in vitro* and *in vivo* by inactivating CDC42-GTP [39], a Rho GTPase which induces the complexing of WASp and the Arp2/3 complex with actin to promote podosome formation. Also, Slit2 can modulate the interaction between Robo1 and WASp in multiple cell types, including smooth muscle cells and neurons [40], [41]. We hypothesized that Slit2N may inhibit iMDDC migration by altering the interaction between Robo1 and key components of the WASp-Arp2/3-β-actin-LSP1 podosome complex. We treated iMDDCs as indicated in Figures 5A and 5B, and after Robo1 immunoprecipitation, examined by Western blot analysis the interactions of Robo1 with WASp, β-actin, and LSP1 (Figures 5A and 5B). The weak interaction between Robo1 and WASp in untreated iMDDCs, and in those treated with M-gp120, or Slit2 negative control + M-gp120, was considerably enhanced after treatment with Slit2N + M-gp120, or with Slit2N alone (Figures 5A and 5B). Robo1 and LSP1 associated very strongly in iMDDCs after treatment with Slit2N alone, with little or no interaction under any other conditions examined (Figures 5A and 5B). There was a moderate interaction between Robo1 and β-actin under all experimental conditions (Figures 5A and 5B).

To ascertain if M-gp120 or Slit2N may affect Robo1's association with LSP1 or WASp by altering the expression level of Robo1 in iMDDCs, we used Western blot analysis. The levels of Robo1 were identical under all conditions examined (Figure 5C). These data indicate that neither M-gp120 nor Slit2N altered Robo1 levels in iMDDCs, however, the IP data (Figures 5A and 5B) suggest that Slit2N may enhance the association of Robo1 with LSP1 and WASp in these cells.

To explore this further, we examined by confocal microscopy the colocalization of Robo1 with WASp and LSP1 in iMDDCs (Figure 5D). In untreated iMDDCs, Robo1, WASp, and LSP1 were diffusely expressed with little or no colocalization (Figure 5D, “UN” panels). When the cells were incubated with M-gp120, they appeared to polarize and to develop a distinct leading edge (Figure 5D, “M-gp120” panels). This morphology is consistent with prior results demonstrating that M-gp120 enhances the migration of iMDDCs ([34], Figures 1A and 1B); however, Robo1 and WASp did not appear to colocalize, and Robo1 and LSP1 did not colocalize to the podosomes at the leading edge of the cells (Figure 5D, “M-gp120” panels). When iMDDCs were treated with Slit2N + M-gp120, and with Slit2N alone, the cells no longer formed one clearly defined leading edge; instead, multiple lamellipodia-like structures were distributed around the periphery of the cells, to which Robo1 strongly colocalized with WASp and LSP1 (Figure 5D, “Slit2N + M-gp120” and “Slit2N” panels). (Note: merged images at a lower magnification are provided in Supporting Information (Figure S2) to demonstrate that the images displayed in Figure 5D are representative of the overall cultures.) We quantitated these colocalization data as previously described. Consistent with the confocal images, colocalization of Robo1 with WASp or LSP1 in untreated iMDDCs was the lowest of all conditions examined, treatment with M-gp120 alone induced little or no enhanced colocalization, and treatment with Slit2N + M-gp120 or Slit2N alone significantly enhanced the colocalization of the aforementioned proteins (Figure 5E).

Taken together, these data suggest that Slit2N may inhibit the M-gp120-induced migration of iMDDCs by enhancing the colocalization of Robo1 with WASp and LSP1. Based on the morphology of the cells, this colocalization of Robo1 with WASp and LSP1 appears to inhibit cellular polarization and the formation of one predominant leading edge, which is consistent with our data demonstrating that Slit2N inhibits the M-gp120-induced migration and transendothelial migration of iMDDCs (Figures 1A and 1B).

### Slit2N Inhibits M-gp120-Induced Src Signaling, and Activation of its Downstream Targets, Pyk2, CDC42, Rac1, and Paxillin

**Figure 6:**
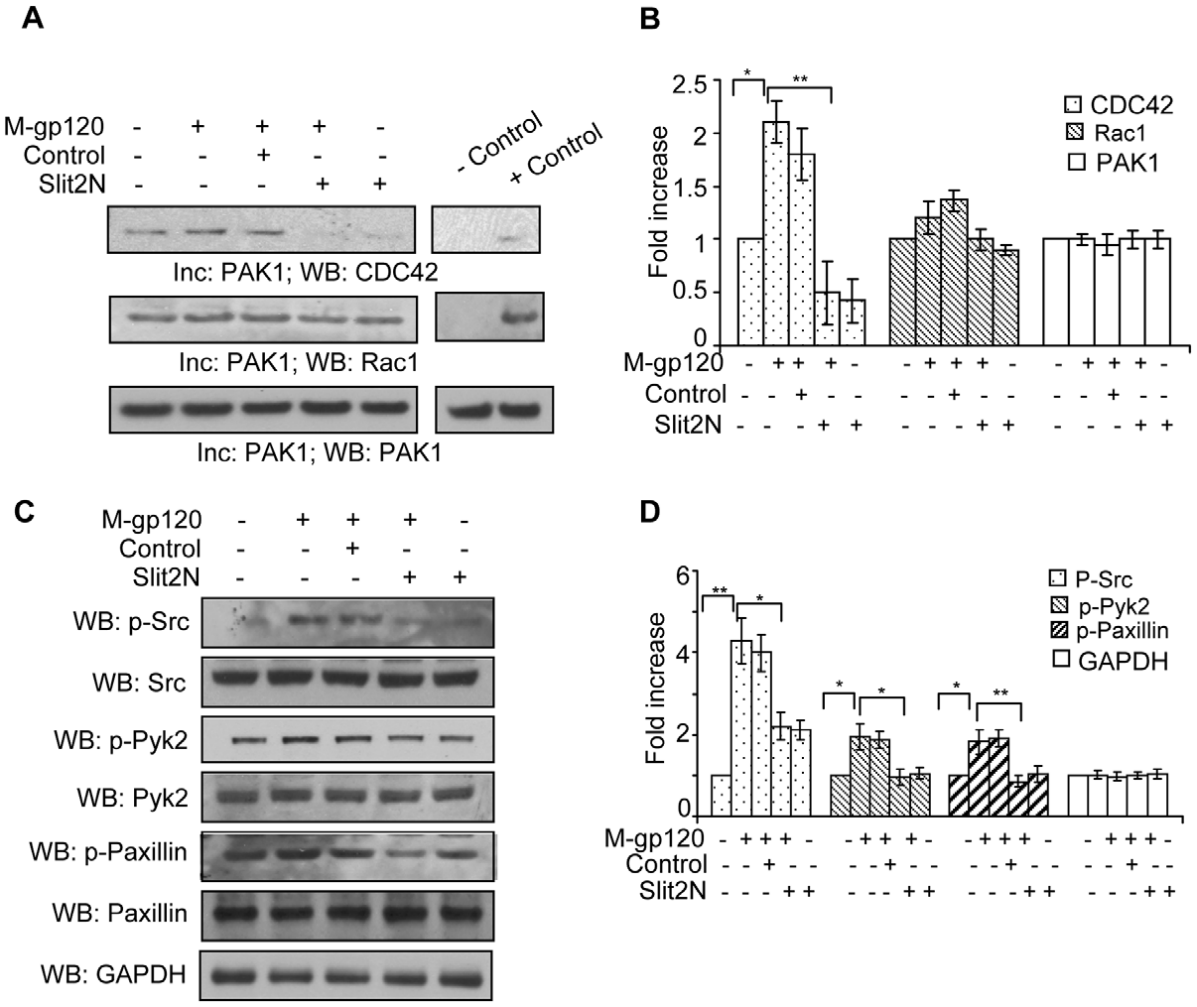
Slit2N inhibits M-gp120-induced signaling through Src, and activation of Pyk2, CDC42, Rac1, and paxillin. (**A**) CDC42 and Rac1 activation assay: Lysates of untreated iMDDCs, and iMDDCs incubated with M-gp120, Slit2 negative control then M-gp120, Slit2N then M-gp120, or Slit2N alone were incubated with activated PAK1-conjugated agarose. Binding of activated CDC42 and Rac1 to PAK1 was visualized by Western blot analysis as described in Materials and Methods. PAK1 used as loading control. Representative assay is shown. (**B**) Quantitative analysis of the CDC42 and Rac1 activation by Western blot analysis. The band intensity in each lane was determined by densitometry. The fold change was determined by calculating the value of each lane vs. the unstimulated control or as indicated. Data represent the mean ± SD of 3 independent experiments (*p<0.05, **p≤0.01). (**C**) Representative Western blot analysis of total and phosphorylated Src, Pyk2 and paxillin in untreated iMDDCs, and in iMDDCs incubated with M-gp120, Slit2 negative control then M-gp120, Slit2N then M-gp120, or Slit2N alone. GAPDH used as loading control. (**D**) Quantitative analysis of phosphorylated Src, Pyk2 and paxillin by Western blot analysis in untreated iMDDCs, and in iMDDCs incubated with M-gp120, Slit2 negative control then M-gp120, Slit2N then M-gp120, or Slit2N alone. GAPDH used as loading control. The band intensity in each lane was determined by densitometry. The fold change was determined by calculating the value of each lane vs. the unstimulated control or as indicated. Data represent the mean ± SD of 3 independent experiments (*p<0.05, **p≤0.01).

Podosomes are required for the migration of iDCs, and other cell types that must travel across tissue barriers [6], [7], [11], [12]. Three primary regulators of podosome formation and disassembly in dendritic cells are Rho GTPase proteins (including CDC42 and Rac1), actin regulatory pathways, and the phosphorylation of receptor tyrosine kinases (including Src and Pyk2) [11], [13]. Robo/Slit interactions can affect migration by modulating Rho GTPases [21], [25]; therefore, we treated iMDDCs as described in Figures 6A and 6B, and examined the activation of CDC42 and Rac1. Since activated CDC42 and Rac1 will bind to PAK-1, we collected cell lysates and incubated them with beads coated with PAK-1. By Western blot analysis we examined the levels of CDC42 and Rac1 that bound to PAK-1. We found that M-gp120 enhanced the basal activation of CDC42, and pretreatment with Slit2N completely inhibited this activation (Figures 6A and 6B). Likewise, Slit2N alone inhibited nearly all of the basal CDC42 activation (Figures 6A and 6B). While the effects were not as pronounced for Rac1, M-gp120 did enhance Rac1 activation, and pretreatment with Slit2N inhibited this activation (Figures 6A and 6B). Src kinase signaling can modulate podosome formation by activating Pyk2 or PI3K [13], and Pyk2 plays a role in the M-gp120-induced migration of dendritic cells [34]. In iMDDCs, we examined total and phosphorylated Src and Pyk2 under the conditions described above. We found that total Src and total Pyk2 levels remained constant under all conditions examined, and M-gp120 enhanced Src phosphorylation, which was inhibited by pretreatment with Slit2N (Figures 6C and 6D). Slit2N also inhibited the M-gp120-enhanced phosphorylation of Pyk2 (Figures 6C and 6D). Src signaling can modulate the activation of paxillin [14], an adhesion-mediating molecule involved in the formation of the podosome ring structure [11]; therefore, we examined paxillin levels under the experimental conditions just described. M-gp120 greatly enhanced the phosphorylation of paxillin in iMDDCs, and pretreatment with Slit2N significantly inhibited this M-gp120-enhanced activation, whereas total paxillin levels remained constant under all conditions examined (Figures 6C and 6D). Taken together, these data suggest that M-gp120 enhances migration and podosome formation in iMDDCs by activating Rac1 signaling through Src and its downstream targets, Pyk2, CDC42 and paxillin.

## Discussion

The ability of iDCs to capture sexually transmitted HIV-1 in the submucosa and localize to lymphatic tissues where they infect CD4^+^ T cells with the virus, depends, at least in part, on the migratory nature of iDCs [3], [4]. Inhibiting the migration of HIV-1-infected iDCs, may limit the dissemination of HIV-1 throughout the host, and the infection of target CD4^+^ T cells.

The HIV-1 envelope protein, M-gp120, which is critical for the attachment and infection of CD4^+^ T cells, can induce the migration of iDCs *in vitro* [34], [42]. This migration likely reflects the chemoattraction of iDCs to HIV-1 *in vivo*. We expanded on these prior data by demonstrating that M-gp120 induced the migration of iDCs through an endothelial cell monolayer, and the formation and localization of podosomes to the leading edge of iMDDCs. Since recent studies suggest a central role for podosomes in the migration of iDCs through tissue barriers [7], enhancing the formation and distribution of podosomes may be a novel mechanism whereby M-gp120 promotes iDC migration.

Podosomes are characterized by a two-part architecture: an actin-rich core and associated proteins, surrounded by a ring-like structure containing proteins such as vinculin and talin, which link integrins to the core [13], [16]. Various components of the podosome core and their interactions have been well-characterized, namely, activation of the Rho GTPase, CDC42, induces the complexing of WASp and the Arp2/3 complex with actin, to promote actin polymerization and podosome formation [38]. Therefore, we examined the effect of M-gp120 on the expression, and cellular localization and colocalization of WASp, Arp2/3 and β-actin in iMDDCs. Consistent with the ability of M-gp120 to induce the migration of iDCs, we found that M-gp120 enhanced podosome formation, their localization to the cells' leading edge, and the complexing of WASp and Arp2/3 with β-actin in the podosomes of migrating iMDDCs. In addition, we identified LSP1, a leukocyte-specific, actin-binding protein [43] that enhances iDC migration by remodeling the actin cytoskeleton [44], [45], as a novel component of the WASp-Arp2/3-β-actin complex in iMDDC podosomes. We also demonstrated that sufficient levels of LSP1 are important for podosome formation and iMDDC migration.

The Slit proteins, which signal when bound to the cognate Robo receptors, were first characterized as developmental neuronal guidance molecules [24]–[27]. They have also been shown to modulate the permeability of lymphatic endothelium [46], and the migration of endothelial cells [47], various cancer cells [48]–[50], and most recently, immune cells, including monocytes [51], neutrophils [20], [21], eosinophils [21], Langerhan cells [22], and dendritic cells [22], [34]. With the exception of eosinophils, whose migration was variously enhanced or inhibited, depending on the specific route of administration [21], Slit2 (when bound to Robo1) inhibited the migration of all types of immune cells examined [20], [22], [51]. Consistent with these data, we found that pretreatment with Slit2N significantly inhibited the M-gp120-induced migration of iMDDCs *in vitro*. Moreover, we determined that the binding of Slit2N to Robo1 inhibited iMDDC migration by preventing the formation and proper intracellular localization of podosomes in M-gp120-treated cells. There was a consistent interaction between Robo1 and β-actin, and treatment with Slit2N enhanced the association of WASp and LSP1 with Robo1.

Podosome formation in leukocytes is primarily regulated by tyrosine kinase signaling through Src, PI3K and Pyk2, which activate various Rho GTPases, including Rac1/2, RhoA and CDC42 [7], [13], [14]. Slit can inhibit the activation of Rho GTPases [21], [25]. In light of recent studies demonstrating the inhibition of immune cell migration by Slit2/Robo1 [19], [20], [22], [51], we hypothesized that Slit2/Robo1 may inhibit iMDDC migration through signaling pathways which modulate podosome formation. Supporting our hypothesis, we observed that Slit2/Robo1 inhibited the M-gp120-induced activation of c-Src, Pyk2, paxillin, Rac1 and CDC42.

**Figure 7:**
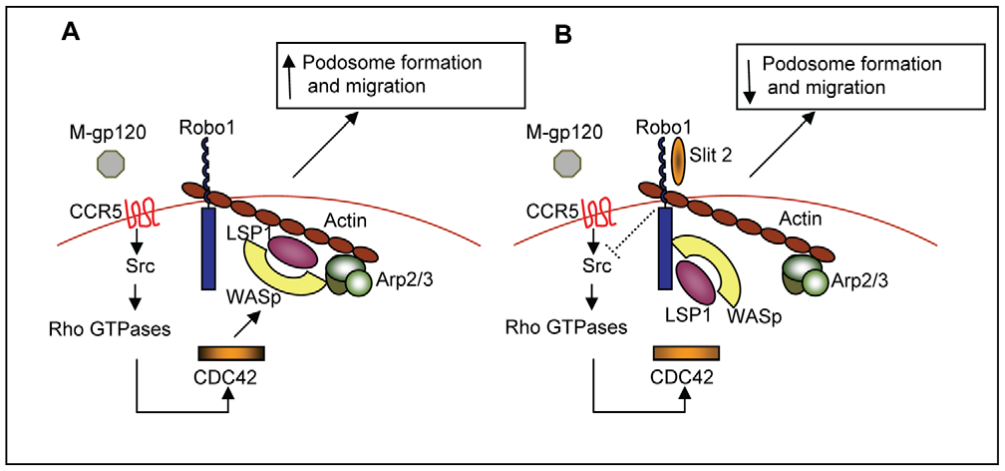
Hypothetical model of the effects of Slit2/Robo1 on HIV-1-gp120-induced podosome formation and migration of iMDDCs. (**A**) HIV-1-gp120 induces podosome formation, and the migration of iMDDCs. (**B**) Slit2/Robo1 inhibit HIV-1-gp120-induced podosome formation and migration of iMDDCs.

Based on these observations, we propose the following model as a potential molecular mechanism by which Slit2/Robo1 may inhibit the HIV-1-gp120-induced migration of iMDDCs (Figure 7). Upon binding to the cell surface receptor CCR5, HIV-1-gp120 induces signaling through c-Src which results in the activation of several GTPases, including CDC42. Activated CDC42 induces the complexing of WASp, LSP1 and the Arp2/3 complex with β-actin, which enhances podosome formation and migration (Figure 7A). When Slit2 binds to Robo1, however, signaling through c-Src and the activation of Rho GTPases, including CDC42, is blocked. Without CDC42 activation, LSP1 and WASp preferentially bind to Robo1, which appears to sequester these proteins away from the WASp-Arp2/3-LSP1-β-actin podosome core complex, and to inhibit podosome formation and iMDDC migration (Figure 7B).

In summary, we found that the interaction between Slit2N and Robo1 inhibited the HIV-1-gp120-induced transendothelial migration of iDCs, a function believed to be key in the initial homing to and internalization of HIV-1 by iDCs, and for the transmission of HIV-1 by iDCs to CD4^+^T cells *in vivo*. Moreover, we demonstrated a strong positive correlation between HIV-1-gp120-induced podosome formation and localization to the leading edge of iMDDCs, with enhanced transendothelial migration. Similarly, when podosome formation, podosome localization, and the colocalization of podosome core proteins were inhibited by Slit2/Robo1 activation, iMDDC migration was inhibited. These findings encourage further studies of the inhibition of dendritic cell migration as a potential clinical strategy to limit the mucosal transmission of HIV-1.

## Materials and Methods

### Cell lines

Human umbilical vein endothelial cells (HUVECs) were purchased from Clonetics, now, Lonza Walkersville, Inc., Walkersville, MD.

### Preparation of Monocyte-Derived Dendritic Cells (iMDDCs) from Blood

Buffy coat was procured from Transfusion Medicine, Children's Hospital, Boston, MA, and from the Blood Transfusion Service, Massachusetts General Hospital, Boston, MA, with strict compliance to the Beth Israel Deaconess Medical Center Committee on Clinical Investigations (CCI) protocol #2008-P-000418. Buffy coat was readily available at these institutions for research purposes, therefore, we did not have to depend on consented donors. In addition, for this work, buffy coat was provided without identifiers. CD14^+^ monocytes were isolated from the buffy coats, using a human CD14^+^ negative selection kit (StemCell Technologies, Vancouver, BC, Canada) per manufacturer's instructions. Monocytes were cultured in complete RPMI media, supplemented with 50 ng/ml human granulocyte macrophage colony-stimulating factor and 50 ng/ml human interleukin-4 (PeproTech, Rocky Hill, NJ). After 6 days, iDCs were characterized by evaluating the expression of DC-SIGN, CD14, HLA-DR, and CD1a with appropriate antibodies using FACS analysis.

### Antibodies and Reagents

The N-terminal, active segment of Slit 2 (Slit2N) and its negative control were prepared as described [36]. M-tropic gp120 was obtained through the NIH AIDS Research and Reference Reagent Program, Division of AIDS, NIAID, NIH. LSP1 antibodies were obtained from BD Transduction Laboratories (San Jose, CA) and Santa Cruz Biotechnologies (Santa Cruz, CA). Vinculin antibody was obtained Sigma Aldrich Corp (St. Louis, MO). Robo1 antibodies were obtained from Abcam (Cambridge, MA) and Sigma Aldrich Corp. WASP, β-actin, Arp2, p-Src, p-Pyk2, p-Paxillin and GAPDH antibodies were obtained from Santa Cruz Biotechnology. Fluorescein isothiocyanate (FITC)–conjugated antibodies CD1a (clone HI149), CD14, and CCR5; phycoerythrin (PE)–conjugated antibodies DC-SIGN and CXCR4; allophycocyanin (APC)–conjugated HLA-DR and CD4; and isotype controls were obtained from BD Pharmingen, San Diego, CA.

### Immunoprecipitation and Western Blot Analysis

iMDDCs were pretreated with Slit2N (1 μg/ml) or Slit negative control (1 μg/ml) for 2 hours in serum-free RPMI 1640, then stimulated with M-gp120 (10 nM) for 2 hours. Cells were then washed, and lysed with RIPA buffer containing protease inhibitors. For immunoprecipitation, equal amounts of protein (0.5 to 1.0 mg) were precleared with protein A-sepharose beads, and incubated with specific antibodies for 2 hours at 4°C. Then, the immune complexes were precipitated with protein A-sepharose beads overnight at 4°C, followed by centrifugation. The beads were washed thrice with RIPA buffer, once with PBS, and suspended in 2X Laemmli buffer. Equivalent amounts of protein extract were run on a 4 to 12% gradient acrylamide gel (NuPAGE Bis-Tris gel; Invitrogen, Carlsbad, CA) and transferred onto nitrocellulose membranes. Immunodetection involved specific primary antibodies, appropriate secondary antibodies conjugated to horseradish peroxidase, and a chemiluminescent Western blotting detection system (GE Healthcare Bio-Sciences, Corp., Piscataway, NJ). For bar graphs, at least three independent Western blots were scanned, and band intensity was measured using Adobe Photoshop CS4. Fold change was calculated by comparing to appropriate controls after subtracting the background.

### siRNA-Mediated Knockdown of LSP1

Small interference RNA-mediated knockdown of LSP1 was performed using SMARTpool duplex RNA oligonucleotides (Dharmacon, Lafayette, CO) with the following four target sequences: GUAACAGUGUGAAGAAAUC, GAAGAGGUAUAAGUUUGUG, AUCAGUGGCUGGAACAAUA, CUAAACCGCUCCAUAGAGA. A non-targeting siRNA (target DNA sequence: AATTCTCCGAACGTGTCACGT) from Qiagen (Valencia, CA) was used as the negative control. siRNAs were transfected into iMDDCs by nucleofection (Lonza Wakersville Inc, Wakersville, MD) per manufacturer's instructions.

### Transwell and Transendothelial Migration Assays

iMDDCs were pretreated with Slit2N (1 μg/ml) or a negative control (1 μg/ml) for 2 hours in RPMI 1640 (0.5% BSA). Approximately 5×10^5^ cells were added to the upper compartment of 24-well transwell chambers (5 μm pores) (Corning Costar Corp, Cambridge, MA). Medium (600 µl), +/− M-gp120 (10 nM) or Slit2N (1 μg/ml), was added to the lower compartments. After 3 hours at 37°C, 5% CO_2_, cells in the lower compartments were counted using a hemocytometer. For the transendothelial migration assay, HUVECs (2×10^5^ cells) were cultured (without stimulation) onto the membrane in the upper compartments of the transwell chambers for 2 days before the start of the experiment. The integrity of confluent HUVEC monolayers was assessed by microscopy. The remainder of the assay was executed as described above, except iMDDCs were incubated for 8 hours before assessing migration. The data are expressed as the percent of cells that migrated to the lower compartments divided by the total number of cells seeded to the top compartments.

### Confocal Microscopy

iMDDCs were cultured on chamber slides. They were pretreated with Slit2N (1 μg/ml) or PBS for 2 hours in RPMI 1640 (0.5% BSA), then incubated with M-gp120 (10 nM) or PBS for 1 hour in RPMI 1640 (0.5% BSA). They were fixed at RT in 4% paraformaldehyde (15 minutes) and blocked with 5% normal goat serum in PBS/Triton (1 hour). Cells were then incubated with an anti-LSP1 monoclonal antibody or anti-Robo1 antibody, and/or antibodies to vinculin, β-actin, WASp and/or Arp2 overnight at 4°C, washed thrice with PBS, and stained with AlexaFluor 568–labeled anti–mouse IgG antibody (Molecular Probes®; Invitrogen) and/or AlexaFluor 488 phalloidin (Molecular Probes) for 2 hours. Subsequently, cells were washed thrice with PBS, and slides were mounted using Prolong Gold antifade with DAPI (4′,6-diamidino-2-phenylindole; Invitrogen). Slides were examined under a Zeiss 510 Meta confocal microscope (Carl Zeiss Microimaging, LLC, Thornwood, NY), and images were acquired using LSM 510 software (Carl Zeiss).

### Percent Area Covered by Podosomes per Cell and Number of Podosomes per Cell

The area covered by podosomes per cell was assessed using the “find object” function of Volocity® software (PerkinElmer, Waltham, MA). The borders of each podosome were drawn by hand. The total area of podosomes, and the percentage area covered by podosomes for individual cells was calculated using Volocity® software. To determine the number of podosomes per cell, the actin cores were identified and counted using the “find objects” function in Volocity®, with verification and correction by eye.

### Quantification of Protein Colocalization

Protein colocalization was quantified using confocal microscopy and Volocity® software. Briefly, the Manders (M) coefficient, which represents the percentage of pixels in the red channel which intersect with signal in the green channel was set to “1” in untreated cells, and fold change in protein colocalization in treated cells was calculated relative to this control.

### CDC42 and Rac1 Activation Assay

CDC42 and Rac1 activation were determined using the Rac/Cdc42 activation assay kit (SGT445, Upstate Biotechnology, Waltham, MA). In brief, cell lysates were incubated with 15 µg/ml p21-activated kinase (PAK)-1 agarose for 1 hour at 4°C, per manufacturer's instructions. Agarose beads were collected by centrifugation, followed by denaturation, boiling of the samples, and SDS-PAGE analysis. Proteins were transferred to nitrocellulose membranes, and Western blot analysis was performed using mouse anti-human CDC42 or Rac1 antibody.

### Statistical Analysis

Results are expressed as the mean ± SD of data from 3 or more independent experiments (as stated), using Student's 2-tailed, paired, t-test. p≤0.05 was considered statistically significant.

## Supporting Information

Figure S1 **Slit2N inhibits M-gp120-induced colocalization of LSP1, WASp, Arp2/3, and β-actin to iMDDC podosomes.** Colocalization of LSP1 with WASp, Arp2/3 and β-actin by confocal microscopy. iMDDCs were cultured on chamber slides and left untreated or incubated with M-gp120, Slit2N then M-gp120, or Slit2N alone (Slit2N incubation: 2 hours; M-gp120 incubation: 1 hour) before fixing and staining cells. Yellow/orange/red  =  merge of LSP1 and WASp, Arp2/3 or β-actin, as indicated; Scale bars  = 5 µm. Representative images are shown.
(TIF)

Figure S2 **Slit2N, but not M-gp120, enhances the colocalization of Robo1 with WASp and LSP1 in iMDDCs.** Colocalization of Robo1 with WASp and LSP1 by confocal microscopy. iMDDCs were cultured on chamber slides and left untreated or incubated with M-gp120, Slit2N then M-gp120, or Slit2N alone (Slit2N incubation: 2 hours; M-gp120 incubation: 1 hour) before fixing and staining cells. Yellow/orange  =  merge of Robo1 and WASp or LSP1, as indicated. Scale bars  = 5 µm. Representative images are shown.
(TIF)
