# Supplementary figures and images for: Slit2N/Robo1 Inhibit HIV-gp120-Induced Migration and Podosome Formation in Immature Dendritic Cells by Sequestering LSP1 and WASp
Source: PLoS One. 2012 Oct 31;7(10):e48854. doi: 10.1371/journal.pone.0048854 (PMC3485365; doi:10.1371/journal.pone.0048854)

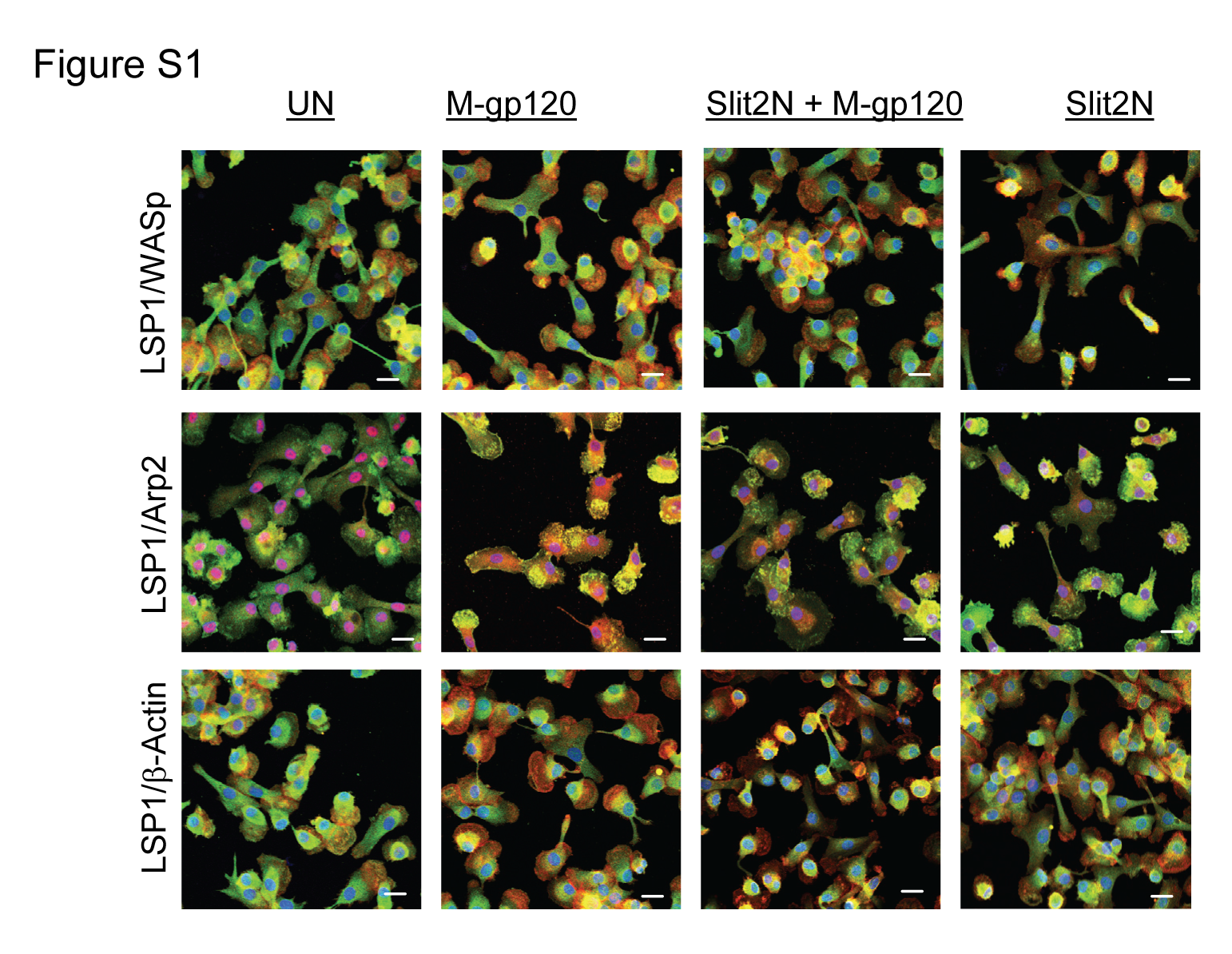

Supplement: Figure S1 — Slit2N inhibits M-gp120-induced colocalization of LSP1, WASp, Arp2/3, and β-actin to iMDDC podosomes. Colocalization of LSP1 with WASp, Arp2/3 and β-actin by confocal microscopy. iMDDCs were cultured on chamber slides and left untreated or incubated with M-gp120, Slit2N then M-gp120, or Slit2N alone (Slit2N incubation: 2 hours; M-gp120 incubation: 1 hour) before fixing and staining cells. Yellow/orange/red = merge of LSP1 and WASp, Arp2/3 or β-actin, as indicated; Scale bars = 5 µm. Representative images are shown. (TIF) [file pone.0048854.s001.tif]

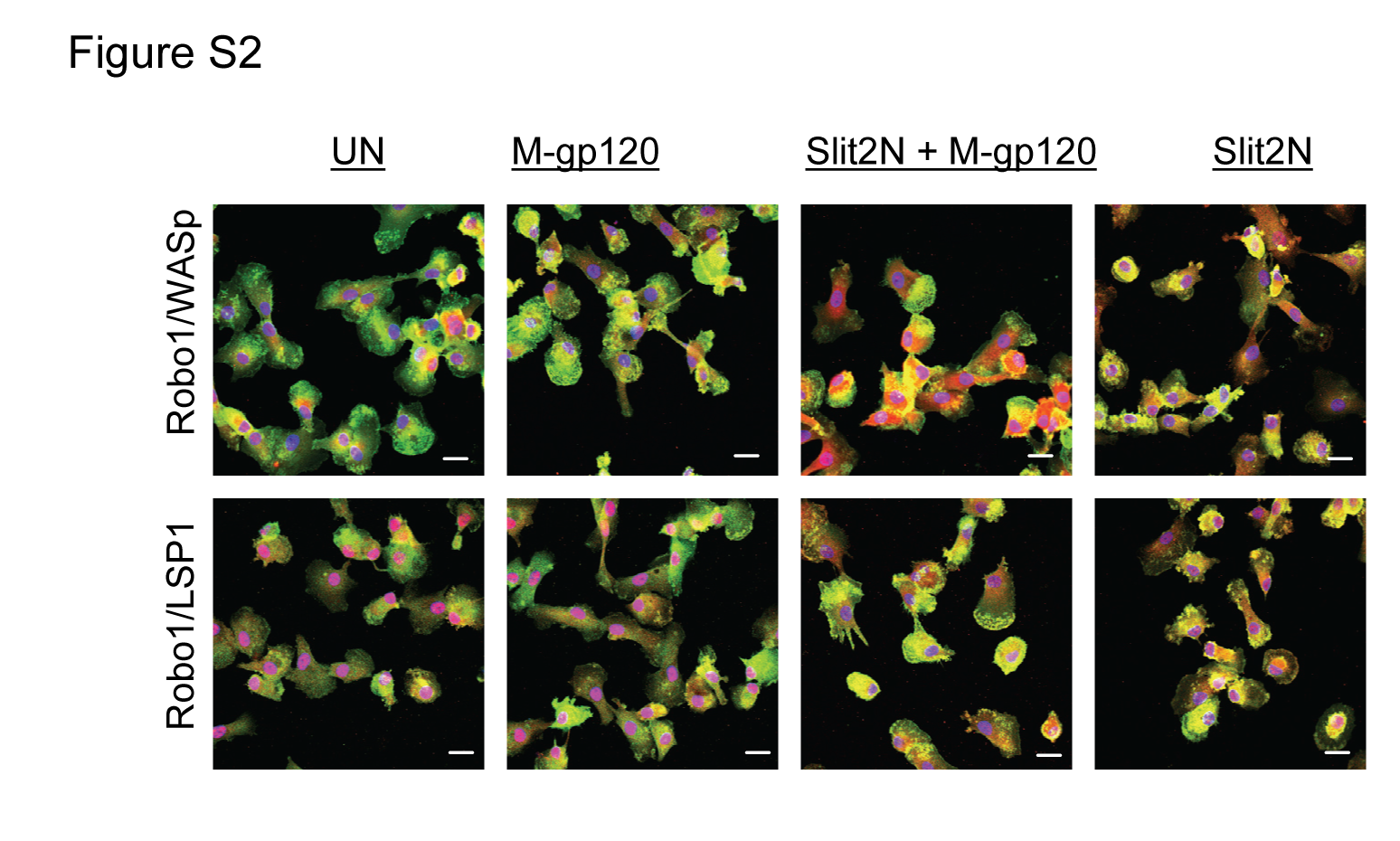

Supplement: Figure S2 — Slit2N, but not M-gp120, enhances the colocalization of Robo1 with WASp and LSP1 in iMDDCs. Colocalization of Robo1 with WASp and LSP1 by confocal microscopy. iMDDCs were cultured on chamber slides and left untreated or incubated with M-gp120, Slit2N then M-gp120, or Slit2N alone (Slit2N incubation: 2 hours; M-gp120 incubation: 1 hour) before fixing and staining cells. Yellow/orange = merge of Robo1 and WASp or LSP1, as indicated. Scale bars = 5 µm. Representative images are shown. (TIF) [file pone.0048854.s002.tif]
